# Tumor-to-tumor metastasis of colon cancer metastasizing to a pancreatic neuroendocrine tumor associated with von Hippel-Lindau disease: a case report

**DOI:** 10.1007/s12328-022-01684-8

**Published:** 2022-08-11

**Authors:** Hiroki Natsui, Junji Kohisa, Seiichi Yoshikawa, Manabu Takeuchi, Ryoma Yagi, Masahiro Minagawa, Tatsuo Tani, Hiroyuki Usuda, Shuji Terai

**Affiliations:** 1 Division of Gastroenterology and Hepatology, Nagaoka Red Cross Hospital, 2-297-1, Sensyu, Nagaoka, Niigata, 940-2085 Japan; 2 Division of Gastroenterology and Hepatology, Niigata City General Hospital, Niigata, Japan; 3 Division of Digestive Surgery, Nagaoka Red Cross Hospital, Nagaoka, Niigata, Japan; 4 Division of Pathology, Nagaoka Red Cross Hospital, Nagaoka, Niigata, Japan; 5 Division of Gastroenterology and Hepatology, Graduate School of Medical and Dental Science, Niigata University, Niigata, Japan

**Keywords:** Tumor-to-tumor metastasis, Pancreatic neuroendocrine tumor, Von Hippel-Lindau disease, Colorectal cancer

## Abstract

Von Hippel-Lindau disease (VHL) is frequently associated with pancreatic neuroendocrine tumors (PNETs). Here, we report a case of tumor-to-tumor metastasis in a VHL patient in whom colon cancer metastasized to the interior of a PNET. A 65-year-old man had undergone bilateral adrenalectomy for pheochromocytomas in both adrenal glands in his 50 s. Genetic screening was performed considering his family history of pheochromocytoma, and he was diagnosed with VHL. PNET was detected, for which the patient was regularly monitored by follow-up imaging. One year ago, the patient underwent right hemicolectomy to remove a tumor in the ascending colon (pT3N0M0, pStage IIA). He was admitted to our department for detailed examination because the pancreatic tumor had grown, and thus, pancreaticoduodenectomy was performed. Diagnostic imaging and histological findings indicated tumor-to-tumor metastasis, in which the patient’s previous colon cancer had metastasized to and proliferated within the PNET. Colon cancer metastasizing to a PNET is extraordinarily rare and has never been reported in the literature. Thus, practitioners should be vigilant for tumor-to-tumor metastasis when performing imaging surveillance of PNETs.

## Introduction

Von Hippel-Lindau disease (VHL) is an autosomal dominant hereditary disease and is frequently associated with pancreatic neuroendocrine tumors (PNETs). PNETs associated with VHL metastasize at a lower rate than typical PNETs; thus, they are typically monitored by surveillance imaging. Tumor-to-tumor metastasis is a phenomenon in which one tumor type serves as the recipient for a second, biologically unrelated, primary donor tumor within the same patient [1]. Here, we report a very rare case of tumor-to-tumor metastasis in a VHL patient whose colon cancer metastasized to the interior of a PNET and was detected during surveillance imaging.

## Case report

**Fig. 1:**
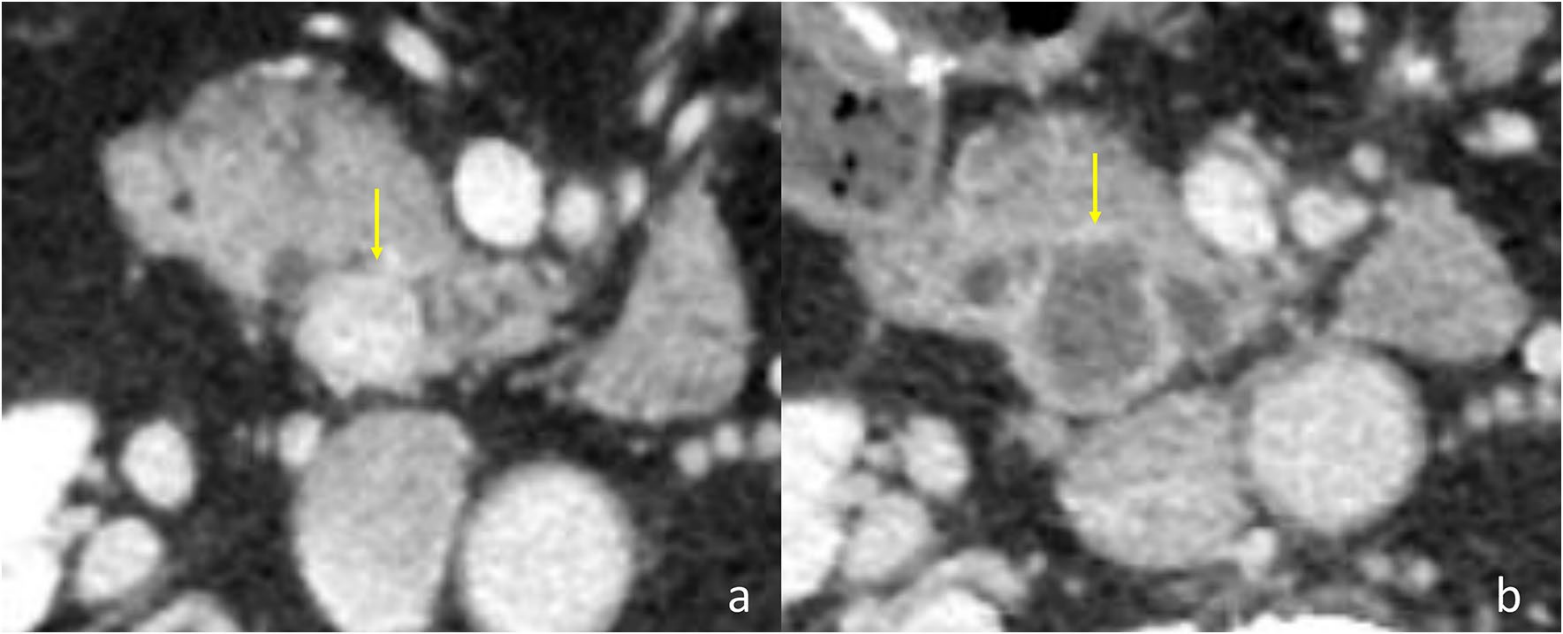
**a**: Contrast computed tomography (CT) imaging showed a 12-mm-sized plethoric tumor in the uncus of the pancreas (arrow). **b**: On admission, Contrast CT showed a 22 mm-sized ring-shaped enhancement tumor in the uncus of the pancreas (arrow)

A 65-year-old man was referred to our department for a detailed examination of a pancreatic tumor. In his 30 s, he underwent distal gastrectomy (B-II reconstruction) for a perforated duodenal ulcer. In his 50 s, he underwent bilateral adrenalectomy to remove pheochromocytomas from both adrenal glands. At this time, genetic screening was performed considering his family history of pheochromocytoma, and he was diagnosed with VHL. A systemic search revealed a 12-mm-sized plethoric tumor in the uncus of the pancreas (Fig. 1a), in addition to spinal hemangioblastomas, which was to be followed up with surveillance imaging. One year before his first visit to our department, he underwent right hemicolectomy to remove the ascending colon cancer (UICC TNM Classification, 8^th^ edition: T3(SS)N0M0, Stage IIA).

**Fig. 2:**
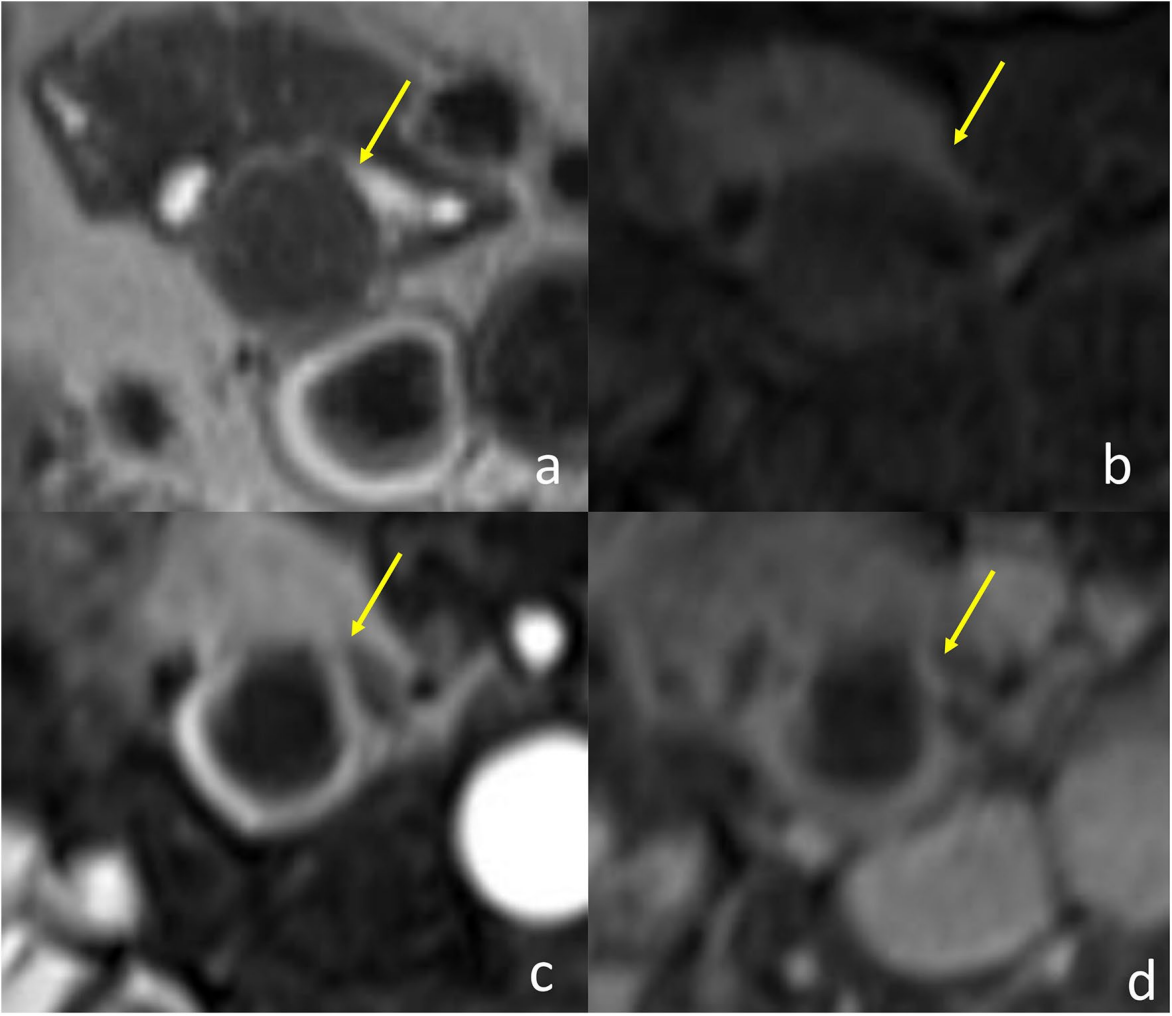
Magnetic resonance imaging (MRI). **a**: T2-weighted MRI showed a hypointense core region (arrow). **b**–**d**: Dynamic contrast-enhanced MRI showed a ring-shaped enhancement that appeared at the margin in early phase images and persisted until delayed phases (arrow) (**b**: plain phase, **c**: early phase, **d**: delayed phase)

**Fig. 3:**
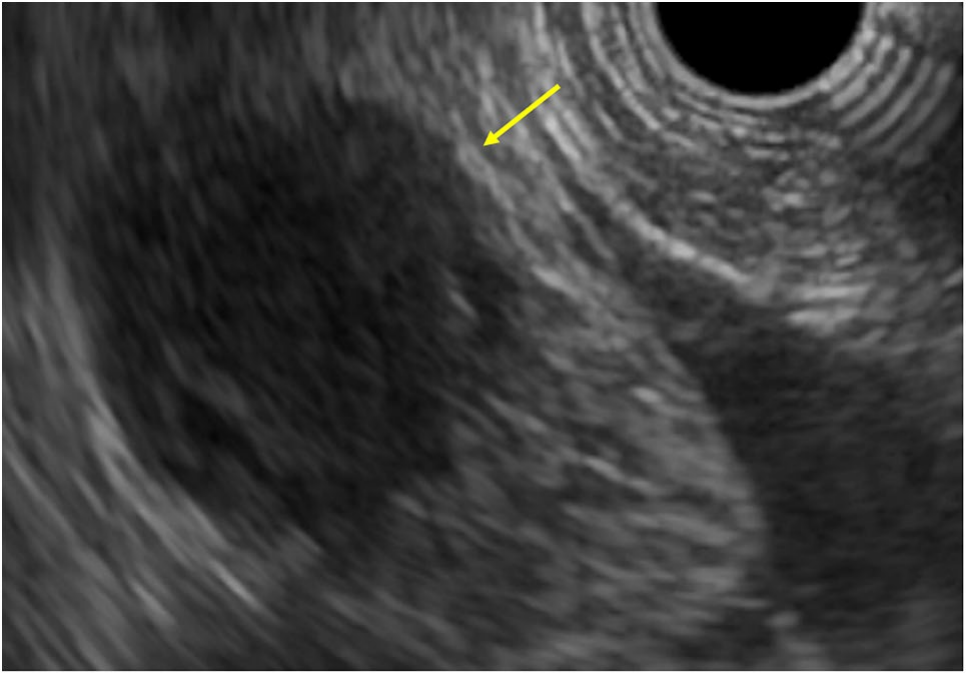
Endoscopic ultrasonography showed a hypoechoic mass with a slightly hyperechoic central component, which were difficult to distinguish (arrow)

**Fig. 4:**
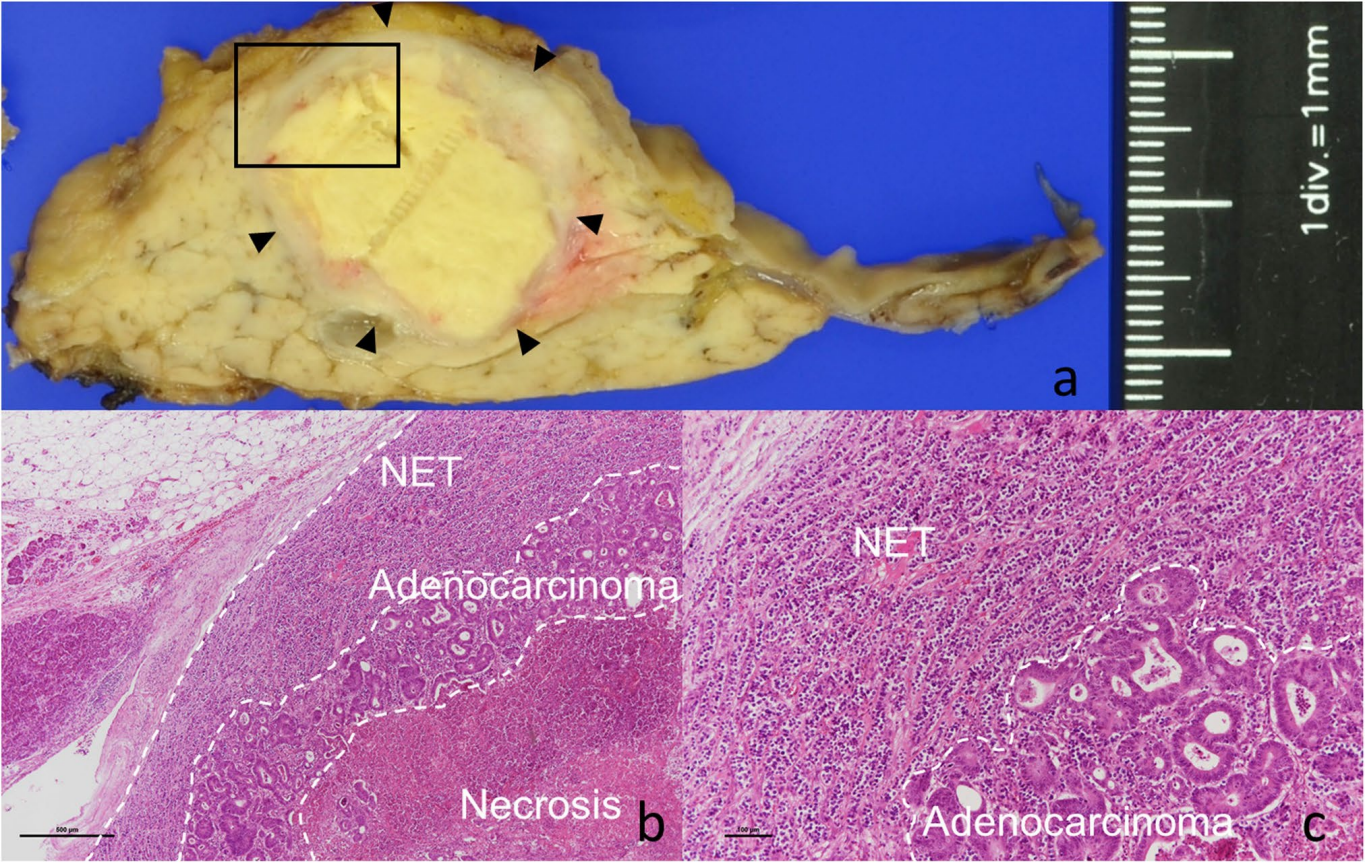
Histopathological findings. **a**: Gross photograph of a pancreatic tumor (arrow heads). Microscopic area is indicated with a square. **b**, **c**: Pathological images of Hematoxylin and Eosin staining showed classic NET cells (G1) of resected pancreas region circumscribed the tumor margin, moderately differentiated adenocarcinoma and signs of necrosis were observed in the interior (magnification. b: × 40, c × 100)

**Fig. 5:**
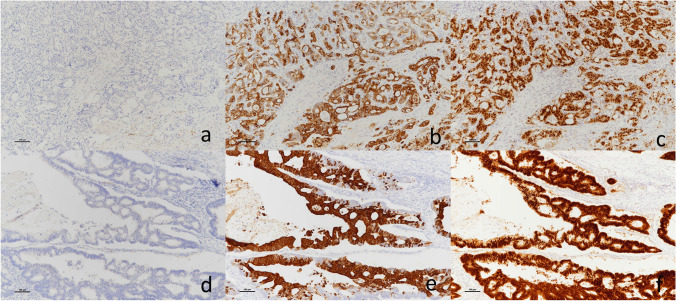
Pathological images of immunohistochemical examination showed that both the adenocarcinomatous region of the pancreas **a**–**c** and the resected colon tumor specimen **d**–**f**- were negative for CK7 and positive for CK20 and CDX2 (**a**, **d**: CK7 staining, **b**, **e**: CK20 staining, **c**, **f**: CDX2 staining) (magnification. a-f: × 100)

**Fig. 6:**
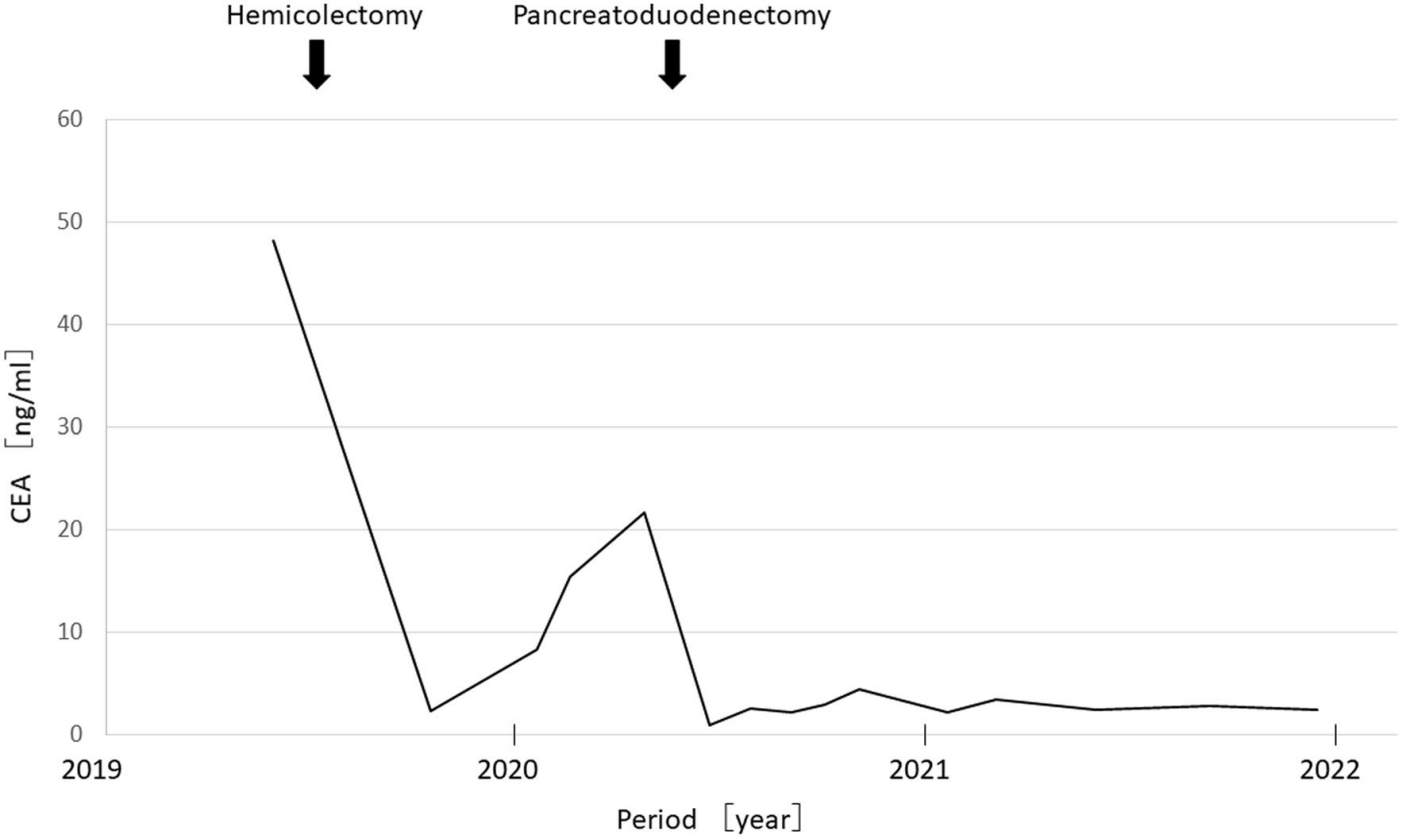
Clinical course. The solid line shows change in serum carcinoembryonic antigen (CEA) levels (ng/ml). CEA normalized after surgery and has remained within reference range since then

He had no symptoms. Blood test results revealed slight elevation in the levels of the tumor marker carcinoembryonic antigen (15.4 ng/mL) but no signs of carbohydrate antigen 19-9 elevation (12.6 U/ml) or abnormal glucose tolerance. Urinary total metanephrines (metanephrine + normetanephrine) were within normal limits. Contrast computed tomography revealed that the well-enhanced tumor in the uncus of the pancreas had grown to 22 mm, and a hypodense ischemic region had developed inside it, showing a ring-shaped enhancement effect (Fig. 1b). This core region was hypointense on T1 and T2-weighted magnetic resonance imaging (MRI); diffusion was restricted at the tumor margin on diffusion-weighted MRI. On dynamic MRI, the tumor interior was poorly enhanced, but ring-shaped enhancement appeared at the margin in early phase images and persisted until later phases (Fig. 2a–d). On endoscopic ultrasonography, the tumor appeared as a hypoechoic mass with a slightly hyperechoic central component (Fig. 3); however, we could not perform endoscopic ultrasonography-guided fine-needle aspiration cytology because the access route could not be secured due to his surgically altered gastrointestinal anatomy. Therefore, we diagnosed the plethoric tumor as a PNET and attributed the ischemic region in its core to intratumoral hemorrhage. Pancreaticoduodenectomy was planned given the tumor’s large maximum diameter (22 mm) with rapid doubling time (415 days). Histological specimens prepared from the resected tissue were examined to establish a pathological diagnosis. While classic NET cells (G1) circumscribed the tumor margin, moderately differentiated adenocarcinoma and signs of necrosis were observed in the interior (Fig. 4a–c). This adenocarcinomatous region had histopathological features similar to specimens prepared from the resected colon tumor. On immunohistochemical examination, both specimens were negative for CK7 and positive for CK20 and CDX2 (Fig. 5a–f). Genetic testing revealed the same KRAS mutation in both tumors (G12V). Based on these findings, the adenocarcinomatous region inside the excised tumor was diagnosed as metastatic colon cancer, representing a rare case of tumor-to-tumor metastasis. After surgery, carcinoembryonic antigen normalized and no recurrence has been detected for more than 21 months (Fig. 6).

## Discussion

VHL is an autosomal dominant hereditary disease characterized by multiple and recurrent neoplastic lesions in a variety of organs. In addition to PNETs, which are estimated to affect 10–17% of patients with VHL, CNS hemangioblastomas, retinal hemangioblastomas, endolymphatic sac tumors, pheochromocytomas, renal cell carcinomas, and pancreatic cysts have been described in VHL patients [2–4].

PNETs associated with VHL metastasize at a lower rate than typical PNETs. In addition, VHL patients often require multiple operations to manage non-neuroendocrine tumors that develop in other organs. For these reasons, practitioners should be cautious when deciding whether surgery is suitable for VHL-associated PNETs [5, 6]. Blansfield, Ganeshan, and others proposed the following criteria as surgical indications for PNETs in VHL patients: (1) tumor size > 3 cm (or > 2 cm for lesions in the head of the pancreas), (2) tumor doubling time < 500 days, (3) mutation in exon 3, and (4) suspicion of regional nodal metastases [4, 7]. After fully explaining our rationale to the patient, we decided to perform pancreaticoduodenectomy because of the tumor’s diameter and doubling rate. Based on diagnostic histopathological examination findings, we identified an adenocarcinomatous region in the inner core of the PNET; based on confirmatory evidence from immunohistochemical and genetic testing, we finally diagnosed it as tumor-to-tumor metastatic colon cancer.

Tumor-to-tumor metastasis has become more widely recognized in recent years. The following four criteria have been proposed as a definition of tumor-to-tumor metastasis: (1) more than one primary tumor must exist within the same patient; (2) the recipient tumor must be a true neoplasm; (3) the metastatic neoplasm should be a true metastasis with established growth within the host tumor and not the result of contiguous growth or embolization of tumor cells; and (4) tumors that have metastasized to the lymphatic system where lymphoreticular malignant tumors already exist are excluded [1, 8]. All these criteria were met in this case.

Cancers that are richly vascularized and have slow growth rates are theorized to be most receptive to tumor-to-tumor metastasis, and renal cell carcinoma, sarcoma, and meningioma are common recipient tumors. Conversely, lung and breast cancers are regarded as the most common donors (i.e., sites of origin) in tumor-to-tumor metastasis [9]. Despite being well-vascularized, PNETs are unlikely to host a secondary tumor because they require immediate surgery. In this case, the patient was scheduled for routine surveillance to monitor his PNET because of his status as a VHL patient; metastasis likely occurred during follow-up.

Only two cases of tumor-to-tumor metastasis to a PNET have been reported; the donor was renal cell carcinoma in both instances [10, 11]. Since renal cell carcinoma is the most common origin of metastatic tumors of the pancreas, it is also considered a likely donor of intratumor metastasis to the PNETs [12–14].

In our case, the donor was the colon cancer, which is unusual. Practitioners engaged in follow-up surveillance of PNET patients should exercise special caution when examining patients who have concurrent breast, lung, or colon cancers or any other malignancies known to frequently metastasize to the pancreas, not just renal cell carcinoma, and should continually monitor their course.

In conclusion, this report describes a very rare case of tumor-to-tumor metastasis in a VHL patient whose colon cancer metastasized to the interior of a PNET. Colon cancer metastasizing to a PNET is extraordinarily rare and has never been reported in the literature. Practitioners should be vigilant for tumor-to-tumor metastasis when performing imaging surveillance of PNETs.
